# 4-Nitro­phthalo­nitrile

**DOI:** 10.1107/S1600536814003468

**Published:** 2014-02-22

**Authors:** Chin Yee Jan, Norzianah Binti Haji Shamsudin, Ai Ling Tan, David J. Young, Seik Weng Ng, Edward R. T. Tiekink

**Affiliations:** a Faculty of Science, Universiti Brunei Darussalam, Jalan Tungku Link BE 1410, Negara Brunei Darussalam; b Department of Chemistry, University of Malaya, 50603 Kuala Lumpur, Malaysia; c Chemistry Department, Faculty of Science, King Abdulaziz University, PO Box 80203 Jeddah, Saudi Arabia

## Abstract

In the title compound, C_8_H_3_N_3_O_2_ (systematic name: 4-nitro­benzene-1,2-dicarbo­nitrile), the nitro group is twisted out of the plane of the benzene ring to which it is attached [O—N—C_ring_—C_ring_ torsion angle = 9.80 (13)°]. In the crystal packing, supra­molecular layers with a zigzag topology in the *ac* plane are sustained by C—H⋯N inter­actions.

## Related literature   

For background to the synthesis of functional phthalocyanines, see: Chin *et al.* (2012 ▶). For a related structure, see: Lin *et al.* (2006 ▶). For the synthesis, see: Rasmussen *et al.* (1978 ▶).
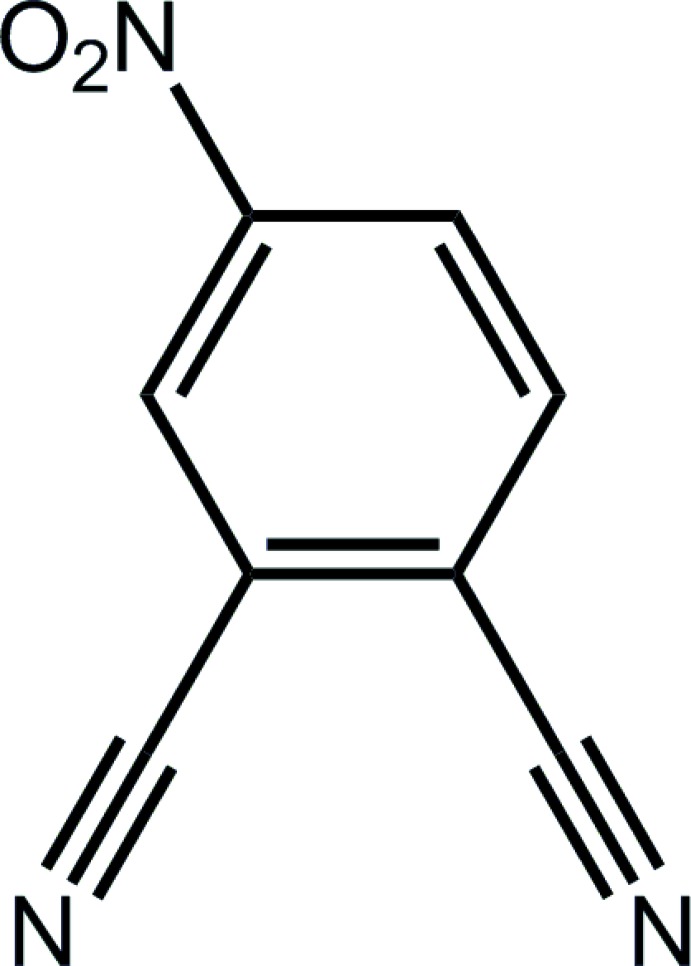


## Experimental   

### 

#### Crystal data  

C_8_H_3_N_3_O_2_
*M* *_r_* = 173.13
Orthorhombic, 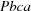

*a* = 12.8642 (3) Å
*b* = 9.2013 (2) Å
*c* = 13.2578 (3) Å
*V* = 1569.29 (6) Å^3^
*Z* = 8
Cu *K*α radiation
μ = 0.94 mm^−1^
*T* = 100 K
0.35 × 0.30 × 0.25 mm

#### Data collection  

Agilent SuperNova Dual diffractometer with an Atlas detector
Absorption correction: multi-scan (*CrysAlis PRO*; Agilent, 2013 ▶) *T*_min_ = 0.626, *T*_max_ = 1.000
7104 measured reflections
1638 independent reflections
1556 reflections with *I* > 2σ(*I*)
*R*_int_ = 0.016

#### Refinement  

*R*[*F* ^2^ > 2σ(*F* ^2^)] = 0.030
*wR*(*F* ^2^) = 0.088
*S* = 1.10
1638 reflections
130 parameters
All H-atom parameters refined
Δρ_max_ = 0.21 e Å^−3^
Δρ_min_ = −0.24 e Å^−3^

### 

Data collection: *CrysAlis PRO* (Agilent, 2013 ▶); cell refinement: *CrysAlis PRO*; data reduction: *CrysAlis PRO*; program(s) used to solve structure: *SHELXS97* (Sheldrick, 2008 ▶); program(s) used to refine structure: *SHELXL97* (Sheldrick, 2008 ▶); molecular graphics: *ORTEP-3 for Windows* (Farrugia, 2012 ▶) and *DIAMOND* (Brandenburg, 2006 ▶); software used to prepare material for publication: *publCIF* (Westrip, 2010 ▶).

## Supplementary Material

Crystal structure: contains datablock(s) general, I. DOI: 10.1107/S1600536814003468/wm5005sup1.cif

Structure factors: contains datablock(s) I. DOI: 10.1107/S1600536814003468/wm5005Isup2.hkl

Supporting information file. DOI: 10.1107/S1600536814003468/wm5005Isup3.cml

CCDC reference: 987296

Additional supporting information:  crystallographic information; 3D view; checkCIF report

## Figures and Tables

**Table 1:** Hydrogen-bond geometry (Å, °)

*D*—H⋯*A*	*D*—H	H⋯*A*	*D*⋯*A*	*D*—H⋯*A*
C2—H2⋯N3^i^	0.962 (14)	2.621 (14)	3.3880 (13)	136.9 (11)
C3—H3⋯N2^ii^	0.950 (14)	2.554 (14)	3.3955 (13)	147.8 (10)
C6—H6⋯N3^iii^	0.943 (13)	2.457 (13)	3.3412 (13)	156.1 (10)

Symmetry codes: (i) 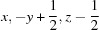; (ii) 
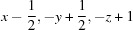
; (iii) 
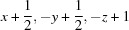
.

**Fig. 1.**
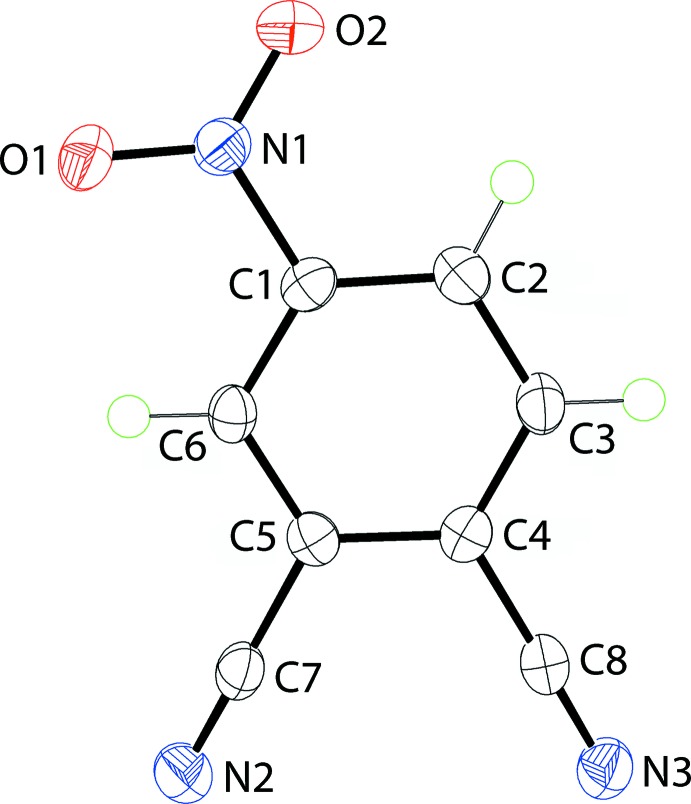
The molecular structure of the title compound showing the atom-labelling scheme and displacement ellipsoids at the 70% probability level.

**Fig. 2.**
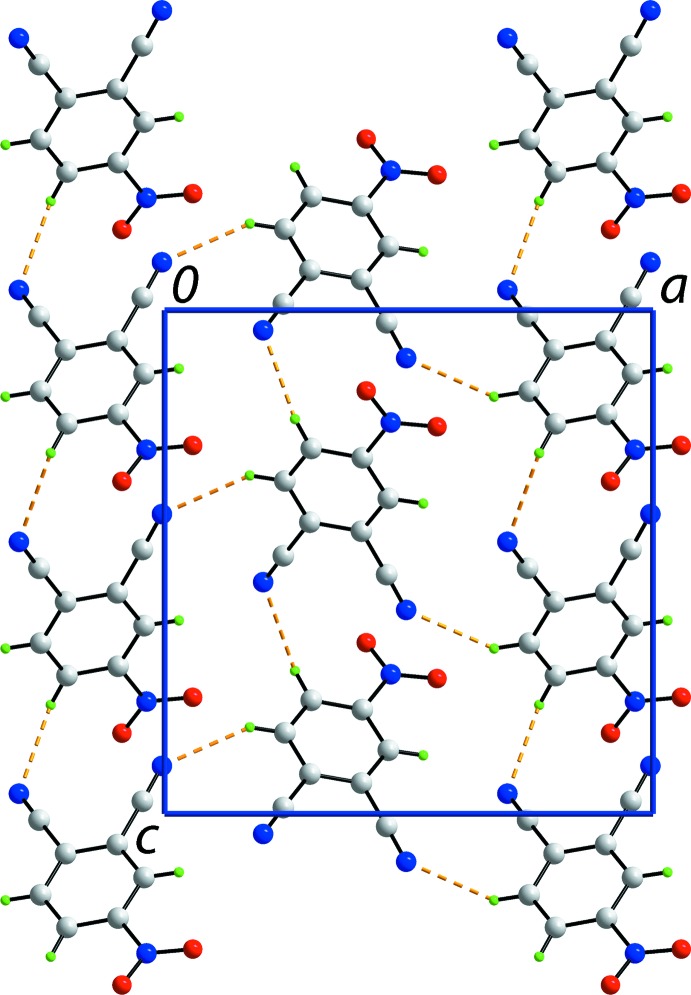
A view of the supramolecular layer in the title compound, sustained by C—H···N interactions shown as orange dashed lines.

**Fig. 3.**
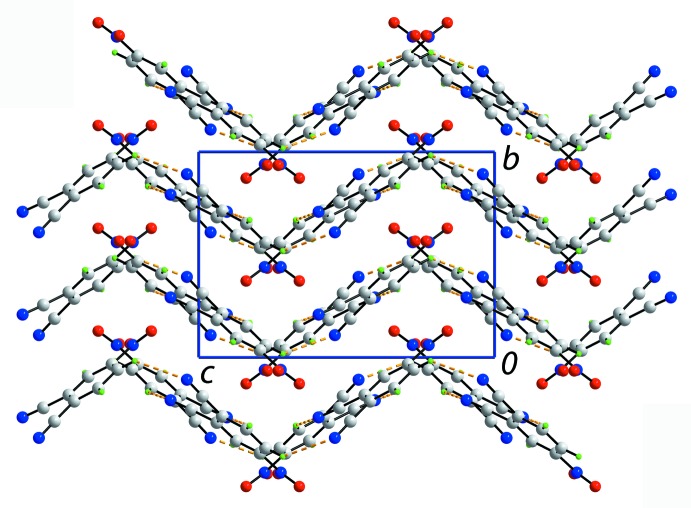
A view of the unit-cell contents of the title compound in projection down the a axis. The C—H···N interactions are shown as orange dashed lines.

## supplementary crystallographic information

### 1. Chemical context

As part of our on-going study of functional phthalocyanines, we have previously reported the synthesis and structure of 4-(prop-2-yn-1-yl­oxy)benzene-1,2-dicarbo­nitrile prepared from 4-nitro­phthalo­nitrile (Chin *et al.*, 2012). We now report the structure of the latter.

### 2. Structural commentary

In the title compound (Fig. 1), the nitro group is slightly twisted out of the plane of the benzene ring to which it is attached as seen in the value of the O1—N1—C1—C6 torsion angle of 9.80 (13)°. A similar small twist was found in the structure of the most closely related compound in the literature, *i.e*. 4-bromo-5-nitro­phthalo­nitrile (Lin *et al.*, 2006).

### 3. Supra­molecular features

Supra­molecular layers (Fig. 2) sustained by C—H···N inter­actions which form 22-membered {···NC_4_N···HC_2_H···NC_3_H···NC_5_H} synthons (Table 1) features in the crystal packing. The layers have a zigzag topolgy and extend parallel to the *ac* plane and stack along the *b* axis (Fig. 3).

### 5. Synthesis and crystallization

The title compound was prepared by a literature procedure (Rasmussen *et al.*, 1978). Thio­nyl chloride (4.3 ml, 0.56 mmol) was added drop wise with stiring over 5 minutes to 4-nitro­phthalamide (2.83 g, 13.5 mmol) in dry DMF (10.4 ml, 0.20 mmol) at 263 to 258 K (salt-ice bath). After 4 h, the homogenous yellow solution was poured onto excess ice-water with vigorous stirring. The precipitate was vacuum filtered, washed with cold water and dried. Crystals for the X-ray study were grown from slow evaporation from its methanol solution. Yield = 1.92 g (68 %), M.pt: 408–413 K (lit. 413–415 K). IR (KBr) ν/cm^-1^: 2924, 2241, 1610, 1587, 1538, 1463, 1356, 1297, 1076, 931, 855, 802, 745, 718.

### 6. Refinement

All hydrogen atoms were refined freely.

### Crystal data

**Table:**

C_8_H_3_N_3_O_2_	*F*(000) = 704
*M**_r_* = 173.13	*D*_x_ = 1.466 Mg m^−^^3^
Orthorhombic, *P**b**c**a*	Cu *K*α radiation, λ = 1.54184 Å
Hall symbol: -P 2ac 2ab	Cell parameters from 4240 reflections
*a* = 12.8642 (3) Å	θ = 3.3–76.1°
*b* = 9.2013 (2) Å	µ = 0.94 mm^−^^1^
*c* = 13.2578 (3) Å	*T* = 100 K
*V* = 1569.29 (6) Å^3^	Prism, colourless
*Z* = 8	0.35 × 0.30 × 0.25 mm

### Data collection

**Table:**

Agilent SuperNova Dual diffractometer with an Atlas detector	1638 independent reflections
Radiation source: SuperNova (Cu) X-ray Source	1556 reflections with *I* > 2σ(*I*)
Mirror monochromator	*R*_int_ = 0.016
Detector resolution: 10.4041 pixels mm^-1^	θ_max_ = 76.3°, θ_min_ = 6.7°
ω scan	*h* = −8→16
Absorption correction: multi-scan (*CrysAlis PRO*; Agilent, 2013)	*k* = −10→11
*T*_min_ = 0.626, *T*_max_ = 1.000	*l* = −16→16
7104 measured reflections	

### Refinement

**Table:**

Refinement on *F*^2^	Primary atom site location: structure-invariant direct methods
Least-squares matrix: full	Secondary atom site location: difference Fourier map
*R*[*F*^2^ > 2σ(*F*^2^)] = 0.030	Hydrogen site location: inferred from neighbouring sites
*wR*(*F*^2^) = 0.088	All H-atom parameters refined
*S* = 1.10	*w* = 1/[σ^2^(*F*_o_^2^) + (0.0522*P*)^2^ + 0.3777*P*] where *P* = (*F*_o_^2^ + 2*F*_c_^2^)/3
1638 reflections	(Δ/σ)_max_ < 0.001
130 parameters	Δρ_max_ = 0.21 e Å^−^^3^
0 restraints	Δρ_min_ = −0.24 e Å^−^^3^

### Special details

**Table:**

Geometry. All e.s.d.'s (except the e.s.d. in the dihedral angle between two l.s. planes) are estimated using the full covariance matrix. The cell e.s.d.'s are taken into account individually in the estimation of e.s.d.'s in distances, angles and torsion angles; correlations between e.s.d.'s in cell parameters are only used when they are defined by crystal symmetry. An approximate (isotropic) treatment of cell e.s.d.'s is used for estimating e.s.d.'s involving l.s. planes.
Refinement. Refinement of *F*^2^ against ALL reflections. The weighted *R*-factor *wR* and goodness of fit *S* are based on *F*^2^, conventional *R*-factors *R* are based on *F*, with *F* set to zero for negative *F*^2^. The threshold expression of *F*^2^ > σ(*F*^2^) is used only for calculating *R*-factors(gt) *etc*. and is not relevant to the choice of reflections for refinement. *R*-factors based on *F*^2^ are statistically about twice as large as those based on *F*, and *R*- factors based on ALL data will be even larger.

### Fractional atomic coordinates and isotropic or equivalent isotropic displacement parameters (Å^2^)

**Table:**

	*x*	*y*	*z*	*U*_iso_*/*U*_eq_	
O1	0.55783 (6)	0.56540 (8)	0.23140 (6)	0.0224 (2)	
O2	0.41285 (6)	0.62811 (9)	0.16017 (6)	0.0284 (2)	
N1	0.46305 (7)	0.56035 (9)	0.22296 (6)	0.0185 (2)	
N2	0.49465 (7)	0.20831 (10)	0.59515 (7)	0.0245 (2)	
N3	0.20184 (7)	0.10850 (10)	0.54001 (7)	0.0214 (2)	
C1	0.40488 (7)	0.46610 (10)	0.29308 (7)	0.0159 (2)	
C2	0.30038 (8)	0.44112 (11)	0.27448 (7)	0.0185 (2)	
C3	0.24639 (7)	0.34933 (11)	0.33907 (7)	0.0183 (2)	
C4	0.29752 (7)	0.28688 (10)	0.42094 (7)	0.0158 (2)	
C5	0.40307 (7)	0.31687 (10)	0.43884 (7)	0.0152 (2)	
C6	0.45807 (7)	0.40675 (10)	0.37365 (7)	0.0156 (2)	
C7	0.45469 (7)	0.25464 (10)	0.52497 (7)	0.0176 (2)	
C8	0.24319 (7)	0.18890 (10)	0.48760 (7)	0.0173 (2)	
H2	0.2670 (11)	0.4827 (16)	0.2163 (10)	0.027 (3)*	
H3	0.1745 (11)	0.3317 (13)	0.3282 (9)	0.020 (3)*	
H6	0.5294 (10)	0.4254 (13)	0.3839 (9)	0.017 (3)*	

### Atomic displacement parameters (Å^2^)

**Table:**

	*U*^11^	*U*^22^	*U*^33^	*U*^12^	*U*^13^	*U*^23^
O1	0.0156 (4)	0.0246 (4)	0.0271 (4)	−0.0022 (3)	0.0032 (3)	0.0031 (3)
O2	0.0248 (4)	0.0300 (4)	0.0304 (4)	−0.0019 (3)	−0.0029 (3)	0.0151 (3)
N1	0.0177 (4)	0.0174 (4)	0.0204 (4)	−0.0004 (3)	0.0013 (3)	0.0015 (3)
N2	0.0198 (4)	0.0303 (5)	0.0235 (5)	0.0001 (3)	−0.0016 (3)	0.0064 (4)
N3	0.0183 (4)	0.0241 (4)	0.0217 (4)	−0.0026 (3)	0.0008 (3)	0.0014 (3)
C1	0.0166 (5)	0.0145 (4)	0.0167 (5)	0.0002 (3)	0.0030 (3)	−0.0003 (3)
C2	0.0176 (5)	0.0204 (5)	0.0174 (5)	0.0014 (3)	−0.0017 (3)	0.0002 (4)
C3	0.0132 (5)	0.0219 (5)	0.0199 (5)	−0.0009 (3)	−0.0010 (3)	−0.0005 (4)
C4	0.0155 (4)	0.0152 (4)	0.0166 (4)	−0.0002 (3)	0.0021 (3)	−0.0022 (3)
C5	0.0152 (4)	0.0144 (4)	0.0159 (4)	0.0021 (3)	−0.0001 (3)	−0.0020 (3)
C6	0.0125 (4)	0.0153 (4)	0.0190 (5)	0.0005 (3)	0.0006 (3)	−0.0021 (4)
C7	0.0135 (4)	0.0185 (5)	0.0208 (5)	−0.0015 (3)	0.0019 (3)	0.0000 (4)
C8	0.0139 (4)	0.0196 (5)	0.0184 (4)	0.0002 (3)	−0.0014 (3)	−0.0023 (4)

### Geometric parameters (Å, º)

**Table:**

O1—N1	1.2252 (12)	C2—H2	0.962 (14)
O2—N1	1.2243 (11)	C3—C4	1.3932 (13)
N1—C1	1.4752 (12)	C3—H3	0.949 (13)
N2—C7	1.1453 (13)	C4—C5	1.4057 (13)
N3—C8	1.1459 (13)	C4—C8	1.4431 (13)
C1—C6	1.3811 (14)	C5—C6	1.3898 (13)
C1—C2	1.3859 (14)	C5—C7	1.4397 (13)
C2—C3	1.3889 (14)	C6—H6	0.943 (13)
			
O2—N1—O1	124.59 (8)	C3—C4—C5	120.44 (9)
O2—N1—C1	117.40 (8)	C3—C4—C8	120.41 (8)
O1—N1—C1	118.01 (8)	C5—C4—C8	119.15 (8)
C6—C1—C2	123.54 (9)	C6—C5—C4	120.23 (9)
C6—C1—N1	117.94 (8)	C6—C5—C7	119.68 (8)
C2—C1—N1	118.52 (9)	C4—C5—C7	120.09 (8)
C1—C2—C3	118.44 (9)	C1—C6—C5	117.66 (9)
C1—C2—H2	120.7 (8)	C1—C6—H6	121.4 (8)
C3—C2—H2	120.8 (8)	C5—C6—H6	120.9 (8)
C2—C3—C4	119.67 (9)	N2—C7—C5	178.04 (11)
C2—C3—H3	119.8 (8)	N3—C8—C4	178.30 (10)
C4—C3—H3	120.5 (8)		
			
O2—N1—C1—C6	−170.25 (9)	C3—C4—C5—C7	178.52 (9)
O1—N1—C1—C6	9.80 (13)	C8—C4—C5—C7	−2.37 (13)
O2—N1—C1—C2	10.12 (13)	C2—C1—C6—C5	0.25 (14)
O1—N1—C1—C2	−169.82 (9)	N1—C1—C6—C5	−179.36 (8)
C6—C1—C2—C3	−1.26 (15)	C4—C5—C6—C1	1.09 (14)
N1—C1—C2—C3	178.34 (8)	C7—C5—C6—C1	−178.85 (8)
C1—C2—C3—C4	0.91 (15)	C6—C5—C7—N2	96 (3)
C2—C3—C4—C5	0.38 (14)	C4—C5—C7—N2	−84 (3)
C2—C3—C4—C8	−178.71 (9)	C3—C4—C8—N3	122 (3)
C3—C4—C5—C6	−1.41 (14)	C5—C4—C8—N3	−57 (4)
C8—C4—C5—C6	177.69 (8)		

### Hydrogen-bond geometry (Å, º)

**Table:**

*D*—H···*A*	*D*—H	H···*A*	*D*···*A*	*D*—H···*A*
C2—H2···N3^i^	0.962 (14)	2.621 (14)	3.3880 (13)	136.9 (11)
C3—H3···N2^ii^	0.950 (14)	2.554 (14)	3.3955 (13)	147.8 (10)
C6—H6···N3^iii^	0.943 (13)	2.457 (13)	3.3412 (13)	156.1 (10)

Symmetry codes: (i) *x*, −*y*+1/2, *z*−1/2; (ii) *x*−1/2, −*y*+1/2, −*z*+1; (iii) *x*+1/2, −*y*+1/2, −*z*+1.
